# A multivariate approach to investigate the associations of electrophysiological indices with schizophrenia clinical and functional outcome

**DOI:** 10.1192/j.eurpsy.2023.2410

**Published:** 2023-05-26

**Authors:** Luigi Giuliani, Nikolaos Koutsouleris, Giulia Maria Giordano, Thomas Koenig, Armida Mucci, Andrea Perrottelli, Anne Reuf, Mario Altamura, Antonello Bellomo, Roberto Brugnoli, Giulio Corrivetti, Giorgio Di Lorenzo, Paolo Girardi, Palmiero Monteleone, Cinzia Niolu, Silvana Galderisi, Mario Maj, Italian Network for Research on Psychoses

**Affiliations:** 1 Department of Psychiatry, University of Campania Luigi Vanvitelli, Naples, Italy; 2 Department of Psychiatry and Psychotherapy, University Hospital, Ludwig Maximilian University of Munich, Munich, Germany; 3 Translational Research Center, University Hospital of Psychiatry, University of Bern, Bern, Switzerland; 4 Psychiatry Unit, Department of Medical Sciences, University of Foggia, Foggia, Italy; 5 Department of Neurosciences, Mental Health, and Sensory Organs (NESMOS), Faculty of Medicine and Psychology, Sapienza University, Rome, Italy; 6 Department of Mental Health of ASL (Local Health Company) of Salerno, Salerno, Italy; 7 Department of Systems Medicine, Psychiatry and Clinical Psychology Unit, Tor Vergata University of Rome, Rome, Italy; 8 Department of Neurosciences, Mental Health and Sensory Organs, Suicide Prevention Center, Sant’Andrea Hospital, Sapienza University of Rome, Rome, Italy; 9 Department of Medicine, Surgery and Dentistry “Scuola Medica Salernitana”, Section of Neuroscience, University of Salerno, Salerno, Italy

**Keywords:** EEG, functional outcome, machine learning, schizophrenia

## Abstract

**Background:**

Different electrophysiological (EEG) indices have been investigated as possible biomarkers of schizophrenia. However, these indices have a very limited use in clinical practice, as their associations with clinical and functional outcomes remain unclear. This study aimed to investigate the associations of multiple EEG markers with clinical variables and functional outcomes in subjects with schizophrenia (SCZs).

**Methods:**

Resting-state EEGs (frequency bands and microstates) and auditory event-related potentials (MMN-P3a and N100-P3b) were recorded in 113 SCZs and 57 healthy controls (HCs) at baseline. Illness- and functioning-related variables were assessed both at baseline and at 4-year follow-up in 61 SCZs. We generated a machine-learning classifier for each EEG parameter (frequency bands, microstates, N100-P300 task, and MMN-P3a task) to identify potential markers discriminating SCZs from HCs, and a global classifier. Associations of the classifiers’ decision scores with illness- and functioning-related variables at baseline and follow-up were then investigated.

**Results:**

The global classifier discriminated SCZs from HCs with an accuracy of 75.4% and its decision scores significantly correlated with negative symptoms, depression, neurocognition, and real-life functioning at 4-year follow-up.

**Conclusions:**

These results suggest that a combination of multiple EEG alterations is associated with poor functional outcomes and its clinical and cognitive determinants in SCZs. These findings need replication, possibly looking at different illness stages in order to implement EEG as a possible tool for the prediction of poor functional outcome.

## Introduction

Despite the continuous advances in pharmacological and psychosocial treatments, schizophrenia still remains one of the most severe mental disorders, characterized by a chronic relapsing course and marked disability in a substantial proportion of patients [1]. Although the reduction of symptoms severity contributes to functional recovery, several studies revealed that subjects with schizophrenia (SCZs) in a chronic stage, with remission of psychotic symptoms, still have serious impairment in different areas of real-life functioning, including independent living, work activities and social relationships [2, 3]. In fact, the functional recovery is influenced by the interaction of multiple factors, which represent major determinants of impairment in the aforementioned real-life functioning areas, beyond psychotic symptoms [4–10].

The identification of objective neurophysiological indices associated with the determinants of functional outcome might represent a crucial step towards the implementation of personalized treatments and the identification of new treatment strategies, aiming at improving the functional recovery of SCZs [11–14]. Indeed, so far, we are not able to predict individual’s outcome across different stages of the illness [15, 16]. In addition, most studies investigating determinants of poor functional outcomes, such as negative symptoms and cognitive impairment, did not contribute to any increase in knowledge concerning the underlying neurobiological processes [17–19].

Identifying biological markers of factors associated with functional outcome, and of the outcome itself, may contribute to the generation of detailed and specific pathophysiological models, resulting in more accurate predictions, as well as to the development of innovative treatment interventions [20].

Electrophysiological (EEG) indices have been largely investigated as possible biomarkers of schizophrenia [21–24].

Several quantitative resting-state EEG and event-related potentials (ERP) alterations have been reported in SCZs in different stages of the illness and many of them are associated with psychopathology, cognitive impairment, and functional outcome [25–29].

In particular, different studies showed that gamma band activity and mismatch negativity (MMN) are associated with functional impairment and may predict the course of the illness in chronic [30–32] and in first-episode psychosis patients, as well as in subjects at clinical high-risk of psychosis [28, 33, 34]. Conflicting evidence has been reported for other EEG bands and ERPs [35, 36]. As to determinants of functional outcome, cognitive impairment was found to be associated with alterations in multiple resting-state frequency bands [29, 37], abnormalities of P300 amplitude and latency [27, 37, 38], deficit in both N100 amplitude and sensory gating [29, 37, 39], and lower MMN amplitude [29, 37, 40–42]. As regard psychopathology, the severity of negative symptoms was found to be related to increased slower rhythms in resting-state recordings and reduced N100 amplitude [25, 43, 44]. Conflicting findings were reported about the relationship between negative symptoms and other ERPs [44].

However, none of these EEG indices has been implemented in clinical practice, probably due to the variability of the methodology across studies (sample size, illness phase, and experimental paradigms) and the paucity of relevant studies investigating several outcome determinants and multiple EEG indices.

Indeed, the majority of the studies focused only on the associations between EEG indices and specific clinical or functional outcome measures, rarely assessing more than one or a few outcome determinants. This represents an important obstacle to the comprehension of the neurobiological mechanisms associated with the outcome of schizophrenia [45]. In fact, as previously reported, the pathways to functional recovery are extremely complex, involving different factors which directly and indirectly influence the real-life functioning of SCZs [4–8]. Recent studies considering candidate EEG biomarkers of schizophrenia and several disease-related variables, such as cognitive impairment and negative symptoms, demonstrated multiple contributions of different EEG indices to cognitive deficits and negative symptoms, leading to poor functional outcomes [45]. In addition, considering that schizophrenia presents a high rate of variability also in terms of pathophysiology [46, 47], the investigation of one or only a few EEG indices, instead of a combination of them, is to limiting for the evaluation of the prognostic value of EEG in schizophrenia. Therefore, the association of these potential EEG markers of schizophrenia with the functional outcome still remains unclear [48]. Lastly, the possibility of implementing EEG indices in clinical routine as prognostic markers of schizophrenia is also related to the ability of formulating outcome predictions beyond group-level prognostication [15, 49].

In order to achieve this goal, in the last decade, different approaches, such as machine learning, deep learning or “multiverse” approaches, were adopted to identify combinations of neurophysiological indices associated with different characteristics of the disease, accounting for the complexity and the heterogeneity of the pathophysiological pathways towards the functional outcome of schizophrenia [21, 50–52]. The multiverse approach indicated no associations among multiple EEG features discriminating patients from controls, suggesting that each feature might subtend a different aspect, thus reflecting the heterogeneity of the syndrome at the phenomenological and pathophysiological level [51]. As a matter of fact, even in the same illness phase, e.g., chronic stage, schizophrenia is characterized by heterogeneity as to the course and functional outcome [5–11].

In light of these observations, our study aimed to identify patterns of EEG indices, among those discriminating SCZs from controls, which might predict the functional outcome of the disease. Therefore, we first identified the EEG markers which best discriminated SCZs from controls, without preselection of the parameters, and then we investigated the relationships of these patterns with the functional outcome and the psychopathological and neuropsychological determinants of the functional outcome, for example, negative symptoms and neurocognitive deficits. We decided to use machine-learning techniques which are able to learn statistical functions from multidimensional data, recognize data patterns, and use those identified patterns to make predictions about individuals [49, 53].

To these aims, we analyzed a well-characterized population of community-dwelling chronic and clinically stable SCZs and matched healthy controls (HCs).

EEGs were recorded in resting-state conditions and during two different tasks, in order to obtain different neurophysiological measures. The EEG indices to analyze as possible prognostic markers of schizophrenia were chosen according to the literature on the topic [23, 29, 37, 54]. Indeed, we selected the neurophysiological indices which have been found to be frequently altered in SCZs and those showing the strongest association with the functional outcome [23, 29, 37, 54]. Therefore, multiple frequency bands and microstates parameters were obtained from the resting-state EEG recording; MMN and P3a were obtained from the EEG recorded during a passive auditory paradigm (in which the subjects had no task), and N100 and P3b were obtained from the EEG recorded during an auditory oddball task. We used a machine-learning approach to identify the EEG patterns which better discriminated SCZs form HCs and we assessed the associations of these patterns with symptom dimensions, cognitive impairment, and real-life functioning in SCZs.

## Materials and methods

### Study participants

The study has been conducted as part of the add-on EEG study of the Italian Network for Research on Psychoses [4–8]. One hundred and forty-eight SCZs and 70 HCs were recruited for the cross-sectional study, at five research sites in Naples, Foggia, Rome “Tor Vergata,” Rome “Sapienza” and Salerno. All 148 SCZs recruited for the cross-sectional study were asked to participate in the longitudinal study, after 4 years of follow-up.

#### Baseline

The group composed of SCZs included individuals consecutively seen at the outpatient units of the five mentioned Italian university psychiatric clinics. Inclusion criteria for SCZs were a diagnosis of schizophrenia according to Diagnostic and Statistical Manual of Mental Disorders, fourth edition (DSM-IV), confirmed with the Structured Clinical Interview for DSM IV – Patient version (SCID-I-P), and an age between 18 and 65 years. HCs were recruited from the community at the same sites mentioned above. The inclusion criterion for HCs was the absence of a current or lifetime Axis I or II psychiatric diagnosis. Exclusion criteria for both groups were: (a) history of head trauma with loss of consciousness; (b) history of moderate to severe mental retardation or neurological diseases; (c) history of alcohol and/or substance abuse in the last 6 months; (d) current pregnancy or lactation; (e) inability to provide informed consent. Other exclusion criteria for SCZs were treatment modifications and/or hospitalization due to symptom exacerbation in the last 3 months. The electrophysiological add-on EEG study was approved by the Ethics Committee of the involved institutions and the study was performed in accordance with the ethical standards laid down in the 1964 Declaration of Helsinki. All participants signed a written informed consent to participate after receiving a detailed explanation of the study procedures and goals.

#### Follow-up

Only SCZs participated in the 4-year longitudinal study. The inclusion criterion of the study was a diagnosis of schizophrenia according to DSM‐IV, confirmed by the SCID‐I‐P. The exclusion criteria of the study were as follows: (a) history of head trauma with loss of consciousness in the 4-year interval between baseline and follow-up; (b) progressive cognitive deterioration possibly due to dementia or other neurological illness diagnosed in the last 4 years; (c) history of alcohol and/or substance abuse in the last 6 months; (d) current pregnancy or lactation; I inability to provide informed consent; (f) treatment modifications and/or hospitalization due to symptom exacerbation in the last 3 months. The longitudinal study was approved by the Local Ethics Committees of the participating centers. All patients signed a written informed consent to participate, after receiving a comprehensive explanation of the study procedures and goals.

### Assessment instruments

#### Baseline

At baseline, all subjects were evaluated for socio-demographic variables such as age, education, and gender, through a clinical form filled out using every available source of information. The Positive and Negative Syndrome Scale (PANSS) was used to rate the severity of positive, negative, and disorganization symptoms in SCZs [55]. Scores for these dimensions were calculated based on the consensus 5-factor solution proposed by Wallwork et al. (for negative dimension we use the Wallwork criteria except for the item “G7––motor retardation,” which was excluded from the calculation of this dimension) [56]. A semi-structured interview, the Brief Negative Symptom Scale (BNSS) was used to assess negative symptoms in SCZs [57]. According to literature [57, 58], the domains evaluated by this instrument loaded on two factors: “experiential domain,” consisting of anhedonia, asociality, and avolition, and “expressive deficit,” including blunted affect and alogia. We also assessed depressive symptoms using the Calgary Depression Scale for Schizophrenia (CDSS) [59] and extrapyramidal symptoms using the St. Hans Rating Scale (SHRS) for Extrapyramidal Syndromes [60]. Neurocognitive functions were rated using the Measurement and Treatment Research to Improve Cognition in Schizophrenia (MATRICS) Consensus Cognitive Battery (MCCB) [61]. This battery assesses seven distinct cognitive domains: processing speed, attention/vigilance, working memory, verbal learning, visual learning, social cognition, and reasoning and problem-solving. Raw scores on the MCCB were standardized to T-scores, corrected for age and gender, based on the Italian normative sample of community participants. For a summary score of cognitive domains including more than one measure and for Neurocognitive and Overall composite scores, we calculated T-score by summing the T-scores of the tests included in each domain and then standardizing the sum to a T-score [62].

We assessed real-life functioning using the Specific Level of Functioning Scale (SLOF), a hybrid instrument that evaluates many aspects of functioning and is based on the key caregiver’s judgment on the behavior and functioning of the patient [63]. It is composed of 43 items and includes the following domains: physical efficiency, skills in self-care, interpersonal relationships, social acceptability, community activities (e.g., shopping, using public transportation), and working abilities. In our study we interviewed the key relative, usually the individual most frequently and closely in contact with the patient.

#### Follow-up

At follow-up, a clinical form was filled with data about the course of the disease and treatment information during the previous 4 years, using every available source of information (patients, relatives, medical records, and mental health workers). All the variables which had been measured at baseline were tested also at follow-up, using the same assessment tools.

### EEG recording procedures

EEGs were recorded only at baseline, using two highly comparable EEG recording systems: EASYS2 (Brainscape, Prague) and Galileo MIZAR-Sirius (EBNeuro, Florence). Before starting the study, a harmonization of the amplifier settings and recording procedure was performed to ensure the same recording settings in all the centers. EEGs were recorded using a cap electrode system with 29 unipolar leads (Fpz, Fz, Cz, Pz, Oz, F3, F4, C3, C4, FC5, FC6, P3, P4, O1, O2, Fp1, Fp2, F7, F8, T3, T4, T5, T6, AF3, AF4, PO7, PO8, Right Mastoid, and Left Mastoid), placed following the 10–20 system. All the leads were referenced to linked earlobes (a resistor of 10 kΩ was interposed between the earlobe leads). A ground electrode was placed on the forehead. The following neurophysiological indices were analyzed: frequency bands activity and microstates extracted from the resting-state EEG recording, four ERP components registered during the two different auditory tasks (MMN, P3a and N100, P3b). Further details on the recording procedure and data preprocessing are provided in the Supplementary materials.

### Statistical analyses

Two sample *t*-test and χ^2^ test were used for group comparisons (SCZs vs HCs). The same analyses were conducted to compare subjects who took part in the longitudinal study with subjects who did not. For the SCZs sample, within-subject comparisons at baseline and follow-up were performed using paired-sample *t*-test and χ^2^ test. Bonferroni-Holm correction was applied to comparisons in order to control for type-I error inflation.

Matlab release 2019b was used for all the above-described analyses.

**Figure 1.**
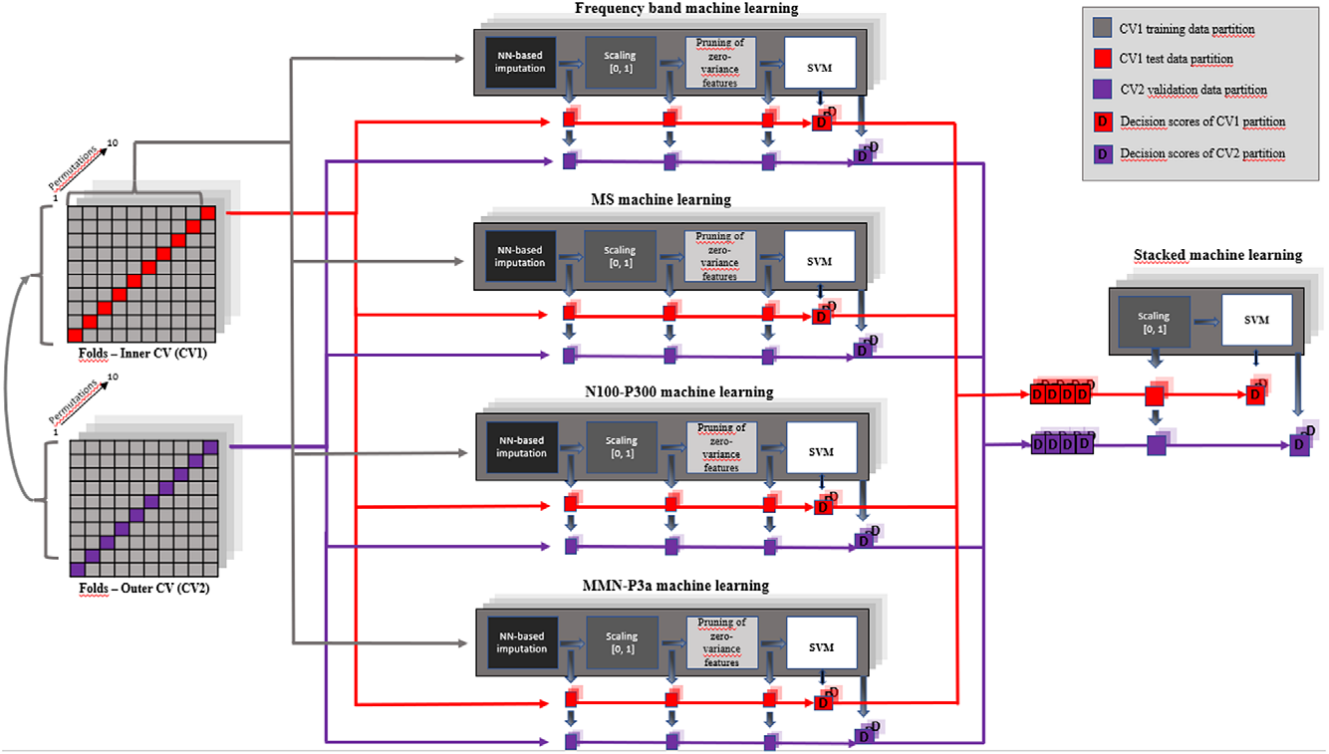
Experimental design of the machine-learning pipelines used to train and cross-validate the unimodal and stacked classifiers. We used nested, repeated cross-validation to train and validate the four individual machine-learning classifiers, consisting of an outer 10-fold cross-validation cycle (CV2), which provided validation participants for computing an unbiased estimate of predictor generalisability to new patients, and an inner 10-fold cross-validation cycle (CV1), which delivered training participants to the multivariate pattern analysis pipeline as well as test participants for features and parameters optimisation. The same nested cross-validation structure was applied to the stacked machine-learning classifier, obtained by combining unimodal classifiers’ outputs within the machine-learning environment. CV, cross-validation; NN, nearest neighbor; SVM, support vector machine.

In order to discriminate SCZs from HCs we generated four different machine-learning classifiers, one for each EEG parameter (frequency bands, microstates, N100-P300 and MMN-P3a) and a global classifier resulting from the combination of the four unimodal classifiers’ output. The machine-learning platform NeuroMiner version 1.0 (https://github.com/neurominer-git; MATLAB release 2019b), was employed to set up a machine-learning strategy for testing the classification performance (SCZs vs HCs) of the four EEG unimodal classifiers and, later, of the global classifier (Figure 1).

The goal of this approach was to investigate whether, using all the information coming from classifiers using different EEG features, could lead to a higher classification accuracy, compared to the single classifiers’ ones. Statistical significance (*p* < 0.05) of individual and global classifiers was assessed with permutation testing, using 1000 permutations of the labels.

The detailed machine-learning pipeline is reported in Supplementary materials and is synthesized in Figure 1.

A *post hoc* analysis was conducted to compare the individual classifier with the best accuracy and the global classifier (McNemar test). *t*-Tests for independent samples were performed for the 10% most frequently selected features of each individual classifier according to the parameter “selection probability,” and Person’s correlations were performed on the same EEG indices to estimate the amount of shared information contained in the variables that were used to distinguish SCZs and HCs. Moreover, we performed a Pearson’s correlation between the chlorpromazine equivalent doses and the output of the global classifier, the classifier’s decision scores, in order to account for the possible impact of antipsychotic medications on the patients’ EEG.

In order to investigate the correlations of the classifiers’ decision scores with illness-related variables and real-life functioning in SCZs at baseline and follow-up, we first projected baseline variables to four factors using a Non-Negative Matrix Factorization (NNMF). We chose NNMF instead of other dimensionality reduction methods because it produces clearly separated and well-defined variance components, enhancing results’ interpretability [64]. The number of factors was chosen to select the optimal dimension that allowed the encoding of data variability while discarding noise. In order to do this, we calculated the variation of the residual error of the data approximation with the variation of the number of estimated components, determining the optimal number of factors by detecting the inflection point of the slope of the reconstruction error [65]. The resulting sparse factor matrices were inspected, and the factors were interpreted according to the variables showing nonnegative loadings on a given factor. After that, we projected the same illness-related variables and real-life functioning indices, measured at 4 years of follow-up, to four factors using the same NNMF algorithm, in order to confirm if the obtained baseline latent variables remained stable from baseline to follow-up. The obtained factor scores were used to compute factor trajectories from baseline to follow-up and paired-sample *t*-test was used to assess the significance of the changes. Pearson’s correlations were performed between classifiers’ decision scores and the scores of each of the four factors resulting from NNMF at baseline and follow-up. All the correlation analyses were corrected for multiple comparisons. Matlab release 2019b was used for NNMF and Pearson’s correlation analysis.

## Results

### Sociodemographic and clinical characteristics of the study sample

**Figure 2.**
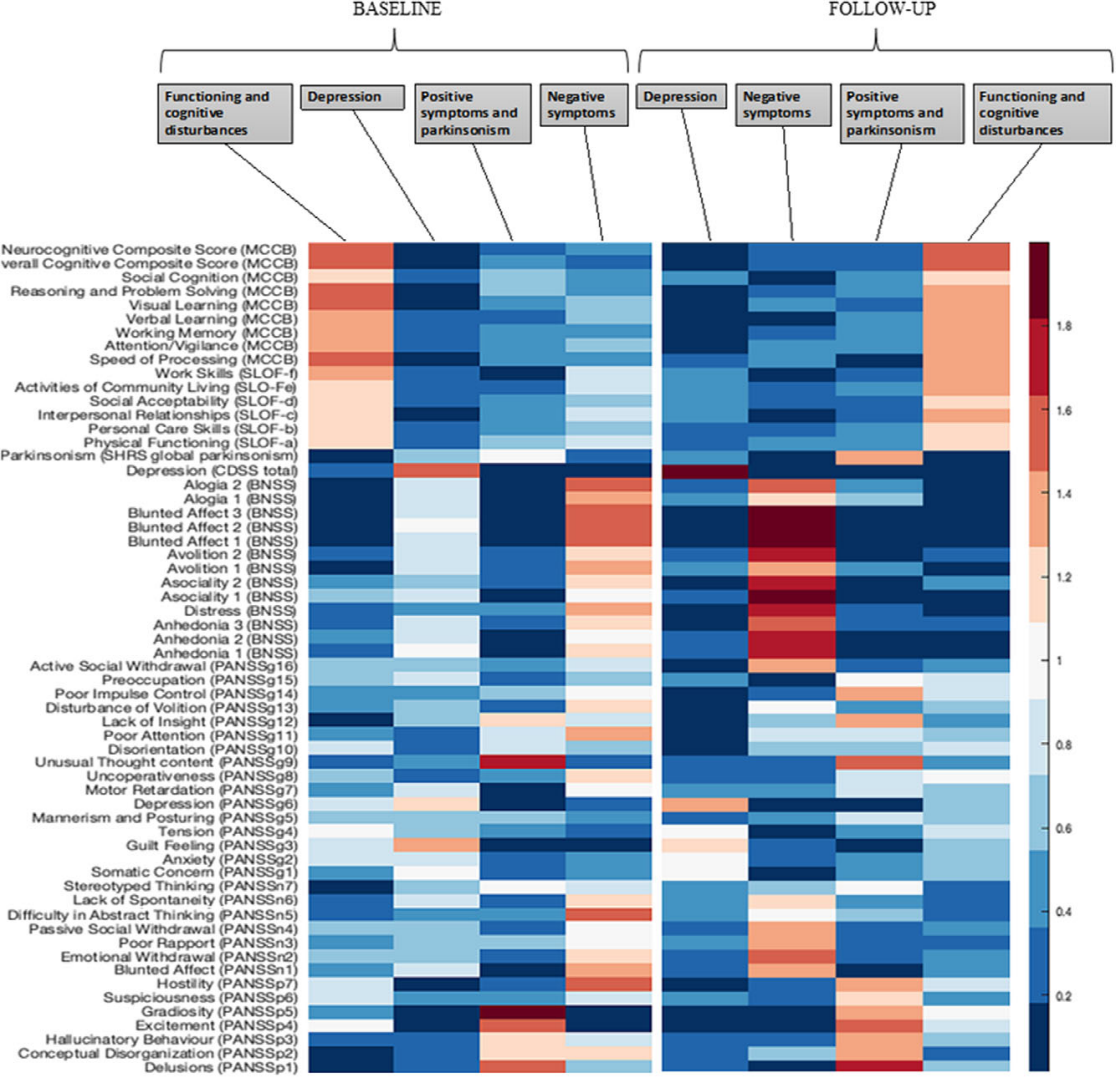
Projection of illness-related and functioning variables, measured at baseline (left) and follow-up (right), to four factors, using Non-Negative Matrix Factorization.

One hundred and forty-eight SCZs and 70 HCs were originally enrolled in the baseline study. Thirty-three SCZs and 13 HCs were excluded because they were found to have more than 25% of missing values in at least one modality data (frequency bands, microstates, MMN & P3a, and N100 & P300). Two subjects from the SCZs group were excluded after visual inspection of the EEG recordings for an excess of artifacts. Therefore, 113 SCZs and 57 HCs were included in the analysis. As regard the EEG recording systems, the EEGs of 88 SCZs (77.9%) and 40 HCs (70.2%) were recorded using the Galileo MIZAR-Sirius system, while the EEGs of the remaining subjects were recorded using the Easys2 system. There was no group difference in the percentage of subjects recorded with the Galileo MIZAR or the Easys2 system (χ^2^ = 1.21; *p* = 0.27). Demographic characteristics and illness-related variables are provided in Table 1. We did not find significant group differences for age (*t* = 1.05; *p* = 0.30). Gender distribution significantly differed between groups (χ^2^ = 7.02; *p* < 0.01), with a higher percentage of males in the patient compared to the control group. Patients had significantly lower education levels than controls (*t* = −3.49; *p* < 0.01). The average duration of illness in the patient group was 12.75 ± 8.29 years. SCZs were characterized by absent to mild positive and disorganization symptom severity (PANSS mean score < 9 for both dimensions) and mild to moderate negative symptom severity (PANSS negative dimension mean score of 15.58 and BNSS total score of 34.88). They had a low mean level of depression (CDSS total score < 4) and Parkinsonism (SHRS Parkinsonism score < 1). SCZs, compared to HCs, showed worse performance on cognitive tests (neurocognitive composite score: *t* = −10.13 and *p* < 0.001 overall composite score including social cognition: *t* = −9.53 and *p* < 0.001) and worse functioning (SLOF-Personal care skills: *t* = −5.40 and *p* < 0.001; SLOF-Interpersonal relationships: *t* = −12.84 and *p* < 0.001; SLOF-Social acceptability: *t* = −5.32 and *p* < 0.001; SLOF-Everyday life skills: *t* = −8.44 and *p* < 0.001; SLOF-Work skills: −9.47 and *p* < 0.001). Sixty-one SCZs from the 113 patients who had taken part in the baseline study, participated in the 4-year follow-up study. Table 2 shows comparisons of demographic characteristics and illness-related variables between follow-up participants (*N* = 61) and the rest of the original SCZs (*N* = 52) sample. Patients who participated in the follow-up study did not significantly differ from the rest of the sample on baseline socio-demographic characteristics and illness-related variables, except for global Parkinsonism (*t* = 3.15; *p* = 0.002) (Table 2). This mean difference in Parkinsonism was relatively small and not clinically significant; thus, the 61 patients participating in the follow-up study can be considered representative of the original sample. The mean values and SDs of all variables included in the analysis at baseline and follow-up are reported in Table 3. In the overall sample of 61 subjects participating in the follow-up study, improvements in the severity of disorganization, the experiential domain of BNSS negative symptoms, and global Parkinsonism were found. Neurocognition was stable, while overall cognitive performance improved after 4 years. We did not find significant changes in real-life functioning from baseline to follow-up. The NNMF analysis showed four stable factors during different time point (baseline and follow-up): one factor captured functioning and cognitive impairments, a second-factor positive symptoms and parkinsonism, a third factor captured negative symptoms, in particular the “expressive deficit” subdomain, and the fourth factor captured depression (Figure 2). Exploring the NNMF factors trajectories, only the factor capturing functioning and cognitive impairment significantly changed (*p* = 0.005) from baseline to follow-up (Supplementary Table S1).

**Table 1.** Socio-demographic, illness-related and real-life functioning variables at baseline.

	HCs (*N* = 57)	SCZs (*N* = 113)	*t*/X2	*p*
Age (mean ± SD)	34.56 ± 12.58	36.34 ± 9.16	1.05	0.30
Gender (M/F)	28/29	80/33	7.02	0.008*
Education (mean ± SD)	14.14 ± 4.15	12.18 ± 3.04	−3.49	<0.001*
Duration of illness (mean ± SD)		12.75 ± 8.29		
PANSS positive (mean ± SD)		7.88 ± 4.31		
PANSS negative (mean ± SD)		15.58 ± 5.96		
PANSS disorganization (mean ± SD)		8.56 ± 3.52		
BNSS total score (mean ± SD)		34.88 ± 16.21		
BNSS—experiential domain (mean ± SD)		21.17 ± 8.81		
BNSS—expressive deficit (mean ± SD)		11.41 ± 7.39		
CDSS total score (mean ± SD)		3.31 ± 4.00		
SHRS—Parkinsonism (mean ± SD)		0.79 ± 1.13		
Neurocognitive composite score (mean ± SD)	51.17 ± 9.98	29.85 ± 12.04	−10.13	<0.001*
Overall composite score (mean ± SD)	49.28 ± 9.29	27.94 ± 11.93	−9.53	<0.001*
SLOF—physical functioning (mean ± SD)	24.85 ± 0.40	24.48 ± 1.08	−2.51	0.01
SLOF—personal care skills (mean ± SD)	34.98 ± 0.13	32.44 ± 3.49	−5.40	<0.001*
SLOF—interpersonal relationships (mean ± SD)	33.87 ± 2.14	23.35 ± 5.88	−12.84	<0.001*
SLOF—social acceptability (mean ± SD)	34.91 ± 0.40	32.27 ± 3.67	−5.32	<0.001*
SLOF—everyday life skills (mean ± SD)	54.80 ± 0.66	46.89 ± 6.86	−8.44	<0.001*
SLOF—work skills (mean ± SD)	28.71 ± 2.10	20.86 ± 5.96	−9.47	<0.001*

Abbreviations: BNSS, Brief Negative Symptom Scale; CDSS, Calgary Depression Scale for Schizophrenia; HCs, healthy controls; PANSS, Positive and Negative Syndrome Scale; SCZs, patients with schizophrenia; SHRS, St. Hans rating scale; SLOF, Specific Level of Functioning Scale.

* Significant *t*‐test after Bonferroni–Holm correction.

**Table 2.** Differences in baseline variables between subjects included and not included in follow-up study.

	FU included (*N* = 61)	FU not-included (*N* = 52)	*t*/X2	*p*
Age (mean ± SD)	36.70 ± 9.16	35.90 ± 9.24	0.46	0.65
Gender (M/F)	43/18	37/15	5.82	0.02
Education (mean ± SD)	12.31 ± 3.00	12.02 ± 3.11	0.50	0.61
Duration of illness (mean ± SD)	12.95 ± 8.58	12.45 ± 7.94	0.30	0.77
PANSS positive (mean ± SD)	8.07 ± 4.80	7.66 ± 3.67	0.49	0.62
PANSS negative (mean ± SD)	15.70 ± 5.57	15.42 ± 6.47	0.25	0.80
PANSS disorganization (mean ± SD)	8.48 ± 3.40	8.66 ± 3.68	−0.27	0.78
BNSS total score (mean ± SD)	34.75 ± 16.22	35.04 ± 16.37	−0.09	0.93
BNSS—experiential domain (mean ± SD)	21.11 ± 9.16	21.24 ± 8.44	−0.08	0.94
BNSS—expressive deficit (mean ± SD)	11.21 ± 7.07	11.65 ± 7.84	−0.31	0.76
CDSS total score (mean ± SD)	3.70 ± 4.07	2.82 ± 3.90	1.16	0.25
SHRS—Parkinsonism (mean ± SD)	1.08 ± 1.26	0.43 ± 0.82	3.15	0.0021*
Neurocognitive composite score (mean ± SD)	29.98 ± 12.88	29.67 ± 10.91	0.13	0.90
Overall composite score (mean ± SD)	28.13 ± 12.36	27.41 ± 10.91	0.24	0.81
SLOF—physical functioning (mean ± SD)	24.61 ± 0.74	24.31 ± 1.39	1.42	0.16
SLOF—personal care skills (mean ± SD)	32.51 ± 3.77	32.35 ± 3.15	0.24	0.81
SLOF—interpersonal relationships (mean ± SD)	23.23 ± 5.71	23.50 ± 6.13	−0.24	0.81
SLOF—social acceptability (mean ± SD)	32.15 ± 3.74	32.42 ± 3.61	−0.39	0.70
SLOF—everyday life skills (mean ± SD)	47.13 ± 6.73	46.60 ± 7.07	0.40	0.69
SLOF—work skills (mean ± SD)	20.26 ± 6.24	21.58 ± 5.57	−1.16	0.25

Abbreviations: BNSS, Brief Negative Symptom Scale; CDSS, Calgary Depression Scale for Schizophrenia; HCs, healthy controls; PANSS, Positive and Negative Syndrome Scale; SCZs, patients with schizophrenia; SHRS, St. Hans rating scale; SLOF, Specific Level of Functioning Scale.

* Significant *t*‐test after Bonferroni–Holm correction.

**Table 3.** Differences in variables measured at baseline and follow‐up.

	Baseline (*N* = 61)	Follow-up (*N* = 61)	*t*/X2	*p*
PANSS positive (mean ± SD)	8.07 ± 4.80	6.54 ± 3.51	2.69	0.009
PANSS negative (mean ± SD)	15.70 ± 5.57	12.74 ± 6.79	2.86	0.006
PANSS disorganization (mean ± SD)	8.48 ± 3.40	6.31 ± 3.30	4.22	<0.001*
BNSS total score (mean ± SD)	34.75 ± 16.22	24.05 ± 16.98	4.21	<0.001*
BNSS––experiential domain (mean ± SD)	21.11 ± 9.16	14.23 ± 9.20	4.77	<0.001*
BNSS––expressive deficit (mean ± SD)	11.21 ± 7.07	8.52 ± 7.38	2.42	0.019
CDSS total score (mean ± SD)	3.70 ± 4.07	2.11 ± 3.31	2.38	0.02
Parkinsonism (mean ± SD)	1.08 ± 1.26	0.52 ± 1.06	3.39	0.001*
Neurocognitive composite score (mean ± SD)	29.98 ± 12.88	33.93 ± 14.80	−2.82	0.07
Overall composite score (mean ± SD)	28.13 ± 12.36	33.66 ± 14.25	−4.14	<0.001*
SLOF––physical functioning (mean ± SD)	24.61 ± 0.74	24.56 ± 0.92	0.32	0.75
SLOF––personal care skills (mean ± SD)	32.51 ± 3.77	32.39 ± 3.45	0.22	0.82
SLOF––interpersonal relationships (mean ± SD)	23.23 ± 5.71	22.48 ± 6.85	0.74	0.46
SLOF––social acceptability (mean ± SD)	32.15 ± 3.74	31.23 ± 4.09	1.45	0.15
SLOF––everyday life skills (mean ± SD)	47.13 ± 6.73	48.31 ± 7.91	−1.23	0.23
SLOF––work skills (mean ± SD)	20.26 ± 6.24	20.85 ± 6.23	−0.67	0.51

Abbreviations: BNSS, Brief Negative Symptom Scale; CDSS, Calgary Depression Scale for Schizophrenia; HCs, healthy controls; PANSS, Positive and Negative Syndrome Scale; SCZs, patients with schizophrenia; SHRS, St. Hans rating scale; SLOF, Specific Level of Functioning Scale.

* Significant *t*‐test after Bonferroni–Holm correction.

### SCZs vs HCs classification performance

Since there was a gender imbalance between the two sample groups (SCZs and HCs), in order to control for the possible confounding effect of this factor, we created a gender classifier, using EEG variables as predictors. We found that this classifier correctly discriminated males from females with a balanced accuracy of 52.6% and was not significant (*p* = 0.25). Moreover, we created a EEG classifier with all the features together entered as input in the algorithm independently from the data modality, including gender among predictors. Thus, we compared this model with an identical classifier without gender among predictors. We found no significant differences in the accuracy of the two classifier (Supplementary Table S2). So, we concluded that EEG indices are not influenced by the gender, and we did not correct the other analyses for this variable. Also education was different between SCZs and HCs, but we did not use it as a covariate in the analyses because lower education level is a well-known consequence of schizophrenia.

**Figure 3.**
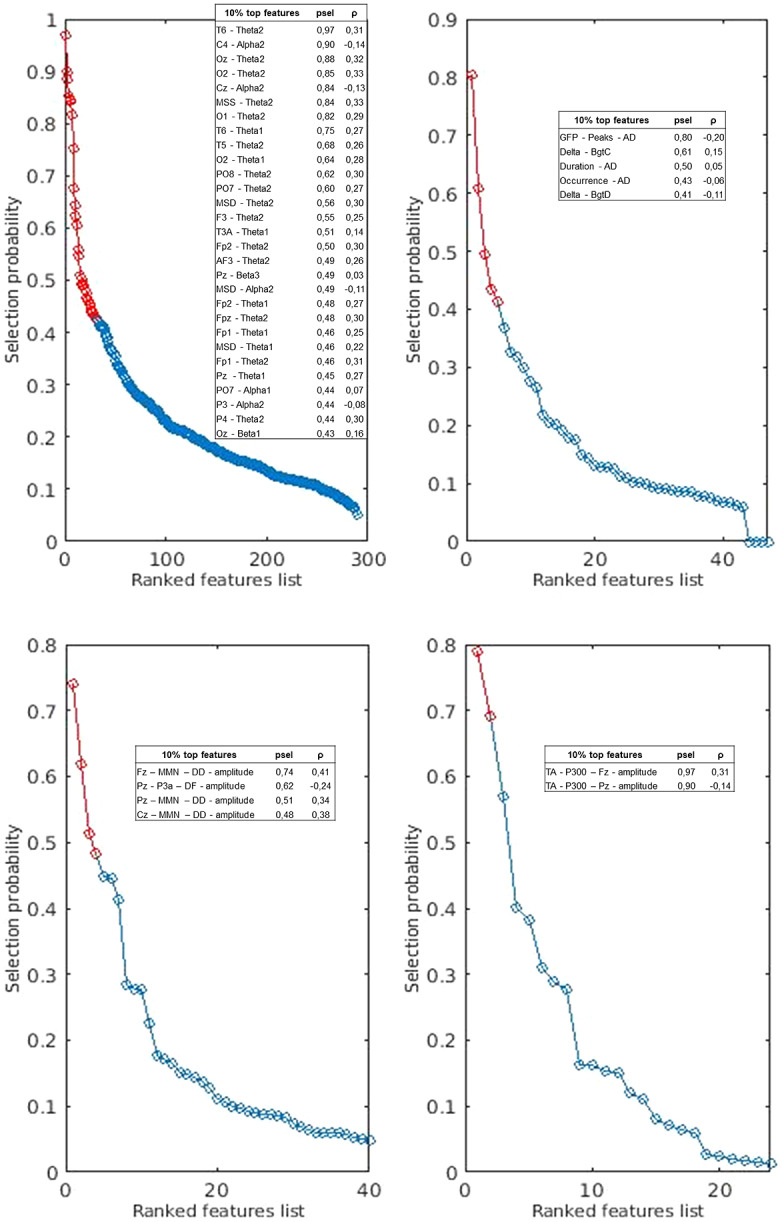
Composition of predictive variable sets selected by the unimodal machine-learning classifiers: frequency bands (A), microstates (B), MMN-P3a (C), and N100-P3b (D). The features were first ranked according to the selection probability measured across all inner-cycle training partitions. Variables ranking among the top 10% of selected features were marked with red and listed with their selection probability (psel) and correlation with the classifier’s outcome (Spearman’s ρ).

**Figure 4.**
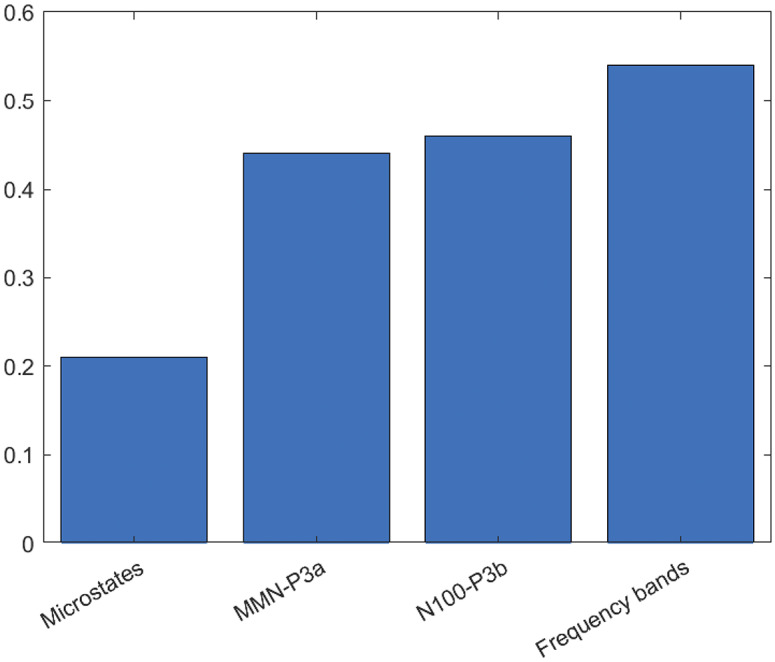
Contribution (Spearman’s ρ) of each individual EEG data modality to the global classifier’s decisions.

As regard to EEG classifiers, detailed statistics of all classifiers are reported in Table 4. The balanced accuracy was highest for the frequency bands classifier and lowest for the microstate one. Figure 3 shows the 10% most frequently selected features for each classifier. The results of the group comparisons on these EEG features and the correlations among these same indices are reported in the Supplementary materials (Supplementary Table S3; Supplementary Figure S1). The global classifier discriminated SCZs from HCs with a balanced accuracy of 75.4% (*p* < 0.01), which was statistically different from the frequency band classifier’s accuracy (χ^2^ = 7.111; *p* = 0.008). As expected, the decisions generated by frequency bands classifier (ρ = 0.54) were the most important for the final classification, followed by N100-P3b (ρ = 0.46) and MMN-P3a (ρ = 0.44). The decision generated by microstates classifier was less important for the classification (ρ = 0.21) (Figure 4).

**Table 4.** Classification performance (SCZs vs HCs) of machine-learning models.

Classification SCZs vs HC	Number of variables	TN	TP	FN	FP	Sensitivity	Specificity	Balanced accuracy	PPV	NPV	NND	PLR	Diagnostic odds ratio	*p*-value
Frequency bands	290	40	82	31	17	72.6	70.2	71.4	82.8	56.3	2.3	2.4	5.9	<0.001
Microstates	43	29	73	40	28	64.6	50.9	57.7	72.3	42.0	6.5	1.3	1.7	0.03
MMN––P3a	40	33	85	28	24	75.2	57.9	66.6	78.0	54.1	3.0	1.8	3.2	0.03
N100––P300	24	38	85	28	19	75.2	66.7	70.9	81.7	57.6	2.4	2.3	5.1	<0.001
Stacking-based classifier	/	40	91	22	17	80.5	70.2	75.4	84.3	64.5	2.0	2.7	7.3	<0.001

Abbreviations: FN, false negative; FP, false positive; NND, number needed to diagnosis; NTN, true negative; PLR, positive likelihood ratio; PPV, positive predictive value; TP, true positive.

We did not find a significant correlation between the chlorpromazine equivalent doses of antipsychotic medications and the global classifier’s decision scores (*r* = 0.160; *p* = 0.171).

### Association of classifiers’ output with illness-related variables and real-life functioning

No significant association was found between the classifiers’ decision scores and the NNMF factors obtained from illness-related variables and real-life functioning measured at baseline. On the contrary, our results showed significant correlations of the global classifier output with depression, negative symptoms, functioning, and cognitive impairment at 4 years of follow-up (Table 5). The direction of the correlations indicated that higher global classifier’s decision score at baseline was associated with more severe negative symptoms, depression and cognitive impairment, and lower real-life functioning at follow-up. The results of the Pearson’s correlations between the individual classifiers’ output and the NNMF factors’ scores at follow-up are reported in Table 5.

**Table 5.** Correlations between classifier decision scores and Non-Negative Matrix Factorization factor scores at follow-up in SCZs.

Classifier’s’ decision scores	Positive symptoms and parkinsonism (r;*p*)	Negative symptoms (r;*p*)	Depression (r;p)	Functioning and cognitive disturbances (r;*p*)
Global classifier	0.014; 0.937	0.399; 0.002*	0.429; <0.001*	−0.332; 0.009*
Frequency bands classifier	−0.018; 0.890	0.271; 0.034*	0.435; <0.001*	−0.229; 0.077
Microstates classifier	−0.132; 0.311	0.092; 0.479	0.282; 0.028	−0.020; 0.880
MMN-P3a classifier	0.120; 0.361	0.341; 0.011*	0.110; 0.399	−0.262; 0.041
N100-P3b classifier	−0.007; 0.955	0.210; 0.104	0.082; 0.530	−0.179; 0.168

Abbreviation: NNMF, Non-Negative Matrix Factorization.

* *p*-value survived correction for multiple tests (*p* < 0.013).

## Discussion

Our results showed that each classifier, using different EEG indices, can identify patterns of neural alterations which are able to significantly distinguish SCZs from HCs at individual level. Combining those patterns of EEG indices recorded under different conditions the classification accuracy significantly increases up to 75.4%. The resulting combination of EEG alterations, in chronic patients with schizophrenia, was associated with real-life functioning and with illness-related variables which have an impact on functional outcomes, such as cognitive impairment, depression, and negative symptoms, at 4-year follow-up [4, 6]. Previous research identified alterations of several EEG indices in SCZs, which are related to different brain functions and associated with different illness features influencing the outcome or with the outcome itself [25–29]. However, despite the results of these studies, no EEG index has been implemented in clinical practice.

In this study, we evaluated multiple EEG indices, recorded under different conditions, and we used a machine-learning approach in order to identify patterns of EEG alterations which could better predict illness outcomes. Using this strategy we tried to improve the precision in detecting the relationships of EEG alterations with clinical features, and the knowledge of the pathophysiological pathways involved in schizophrenia outcome. Indeed, schizophrenia is a heterogeneous syndrome with a high variability in brain structure influenced by gene–environment interactions [66–68]. Moreover, the pathways towards the outcome are extremely complex, with several factors influencing real-life functioning of people with schizophrenia [4–6, 8, 69]. A combination of factors more than any single of them is probably involved in determining individual subject’s outcome, and the identification of reproducible, objective indicators might facilitate the implementation of translational studies results, improving the knowledge about the relative pathophysiological mechanisms. Previous studies used different approaches to investigate multiple EEG alterations in schizophrenia and the correlations between these neurophysiological alterations and illness-related variables [51, 70, 71]. The majority of these studies demonstrated that a weighted combination of EEG features provides better information about the characteristics of the disorder than any single index. However, only a limited number of parameters for each EEG index were included and varied among studies. Within this framework, machine-learning methods have the advantage of learning statistical functions from multidimensional data in order to make prediction about individuals. Therefore, in this study they allowed us to recognize, among a huge amount of parameters (e.g., band activity or ERP amplitude at multiple electrode sites) of different markers, an EEG pattern that was able to discriminate single SCZs from controls. Furthermore, the summary index of this EEG pattern, represented by the decision scores of the global classifier, could be used to investigate the association of such specific combination of neurophysiological markers with the functional outcome, as well as with the clinical and neuropsychological determinants of functional outcome. Indeed, we found that the most selected features by each classifier were poorly correlated to each other, except for the microstates parameters which were significantly associated with theta and alpha activity. These results, in line with those obtained with the multiverse approach [51] demonstrate that combining multiple EEG parameters associated with different characteristics of the disease could lead to a better recognition of the heterogeneous pathophysiological mechanisms, allowing more accurate predictions of the SCZs outcome.

Among the different EEG indices investigated in this study, resting-state frequency bands activity turned out to be the most important feature for the classification of SCZs and HCs, while microstates parameters seem to be redundant with the frequency bands oscillations, adding very little information to the global classifier. According to previous findings, we found that slower band activity alterations were the most specific of schizophrenia, and, in particular, decreased alpha 2 activity and increased theta 1 and theta 2 activity [23]. The alterations in theta and alpha activity are associated with gray and white matter volume reduction in SCZs. Theta activity is associated with learning and its alterations are present in first-degree relatives of SCZs, are independent of antipsychotic medications, and are associated with biological vulnerability to schizophrenia [72, 73]. Genetic analyses showed that theta activity is correlated with two different genetic components, comprising genes participating extensively in brain development, neurogenesis, and synaptogenesis [74]. Theta abnormalities were also mediated by gene clusters involved in glutamic acid pathways, cadherin, and synaptic contact-based cell adhesion processes. Alpha rhythm is functionally related to memory and attention [75], and is associated with the default mode network activity, involved in cognitive functioning [18]. Some genome-wide and positional gene-based analyses showed correlations between alpha activity and tissue-specific single nucleotide polymorphism (SNP), codifying for protein involved in signal transmission, inflammation, and other biological functions [76]. These associations were found principally at the cortical level (hippocampus, frontal cortex, anterior cingulate cortex) and in putamen [76]. According to these findings, it is possible to assume that slower band activity in SCZs reflects alterations of cortical functions linked to specific genetic patterns.

Correlation analyses revealed that the global classifier’s decision scores were associated with real-life functioning and different illness-related variables (cognitive impairment, depression, and negative symptoms) at follow-up. On the opposite, we did not find any association between positive symptoms and disorganization. Negative symptoms and cognitive impairment are core features of schizophrenia, and are present, respectively, in more than 50 and 80% of patients [77, 78].

Available evidence indicates that, differently from positive symptoms and disorganization, they are largely present at the onset of the disorder and during the prodromal stages of the disease [77, 79]. Moreover, in more than half of the cases, negative symptoms have a continuous or relapsing course and the cognitive deficit is relatively stable throughout the course of the illness, unlike positive symptoms, which usually have variable severity [77, 80]. Both cognitive dysfunction and negative symptoms are associated in chronic patients with poor functional outcome [4–8, 81].

No correlations were found with the same features measured at baseline. Our hypothesis is that neurophysiological alterations occur before their related clinical manifestations and reflect the severity of these manifestations measured months or years after the neurophysiological findings.

The study has a number of limitations. The first one is the sample size, which is larger compared to previous EEG studies, but relatively small considering the complexity of the machine-learning structure. Moreover, in order to make our findings more generalizable, the above-reported classifiers should be applied to an independent sample. Additionally, the study sample is composed only of SCZs and HCs. In order to improve the specificity of the EEG model, it is necessary to include also patients with other psychiatric syndromes. Moreover, our sample is composed of chronic patients, with an average duration of illness of 12.75 years and a median age of 36.34 years. Schizophrenia is particularly prevalent in young adults between 20 and 30 years of age and the onset follows years of prodromal symptoms and leads to disability in about half of the patients [82]. Furthermore, different studies demonstrated that the early intervention leads to a better prognosis [83, 84]. Therefore, the main goal of any prognostic tool should be the early recognition of the illness and the possibility to make outcome predictions at the onset of the syndrome. To do this, our model needs to be tested also in first-episode psychotic and at-risk subjects. Furthermore, the prognostic information obtained from the analysis does not allow making predictions about individuals, but it only describes the associations between electroencephalographic patterns and outcome measures at a group level.

These results suggest that a combination of different EEG alterations found in SCZs and associated with the main determinants of functional outcome and the outcome itself could be able to predict the course of schizophrenia. To assess whether this neurophysiological pattern can be implemented as a prognostic marker of schizophrenia in clinical practice, further studies are required including validation samples and subjects at different stages of the disorder.

## Data Availability

All data generated or analyzed during this study are included in this published article.

## Supplementary material

For supplementary material accompanying this paper visit http://doi.org/10.1192/j.eurpsy.2023.2410. click here to view supplementary material
