# Supplementary material for: A multivariate approach to investigate the associations of electrophysiological indices with schizophrenia clinical and functional outcome
Source: Eur Psychiatry. 2023 May 26;66(1):e46. doi: 10.1192/j.eurpsy.2023.2410 (PMC10304998; doi:10.1192/j.eurpsy.2023.2410)
Supplement: Supplementary file 1 [file S0924933823024100sup001.docx]

**A multivariate approach to investigate the associations of electrophysiological indices with schizophrenia clinical and functional outcome**

Luigi Giuliani, Nikolaos Koutsouleris, Giulia Maria Giordano, Thomas Koenig, Armida Mucci, Andrea Perrottelli, Anne Reuf, Mario Altamura, Antonello Bellomo, Roberto Brugnoli, Giulio Corrivetti, Giorgio Di Lorenzo, Paolo Girardi, Palmiero Monteleone, Cinzia Niolu, Silvana Galderisi, Mario Maj, Italian Network for Research on Psychoses

**Supplementary materials**

**eMethods**

**eResults**

**eTables**

**eFigures**

**eReferences**

1. **eMethods**
   1. EEG recording procedures

Participants were instructed not to drink coffee or tea and to abstain from smoking cigarettes in the 2 hours before the beginning of the recording session and did not take psychotropic medications in the morning. Information on the quality of sleep during the night prior to the recording was collected and the EEG session was postponed if the subject reported a non-restoring sleep. Subjects were seated in a reclining chair, in a sound attenuated room, minimizing eye movement or muscle tension. For resting-state EEG recording, EEG was continuously recorded for 5 min while the subjects were at rest with closed eyes. ERPs were recorded during two tasks. The N100-P3b were recorded during an auditory “odd-ball” task, after the presentation of 320 standard stimuli (1500-Hz, 80 dB) and 80 target stimuli deviant for their frequency (1000-Hz, 80 dB). Patients were asked to press the button as fast as possible upon the appearance of every target stimulus. Participants who scored <60% on the behavioral target detection task were excluded from the analysis. MMN-P3a were recorded during the presentation of 2400 tones (80 db SPL), of which 83.3% were standard tones (50 msec, 1000 Hz), 8.3% duration (d) deviant tones (100 msec, 1000 Hz) and 8.3% pitch (p) deviant tones (50 msec, 1200 Hz) with an interstimulus interval of 450 msec. During the presentation of stimuli subjects were asked to watch a silent animated cartoon, and after the paradigm ended, they were asked some questions regarding the video.

All impedances of the leads were kept below 5 kΩ. The EEG data were filtered with a band-pass of 0.15–70 Hz and the sampling rate was 512 Hz. A calibration was performed for all channels, using a 50 μV sine wave, before each recording session. In order to check for artifacts, a horizontal electro-oculogram (hEOG) from the epicanthus of each eye and a vertical EOG (vEOG) from the leads beneath and above the right eye were also recorded.

- 1. EEG data preprocessing

Experts from the coordinating center (Naples) using Brain Vision Analyzer software (Brain Products, Munich, Germany) performed all the analyses on data collected by the different recording sites. Data with high line noise were excluded from further analysis. Eye movements and eye blinks were corrected using independent component analysis (using a specific function of the Brain Vision Analyzer software), while body movements, muscle activity and technical artifacts were discarded manually through visual inspection during centralized data preprocessing for artifacts.

For ERPs analyses data were parsed into epochs of 1000 msec duration, which were time-locked to the onset of the stimuli and spanned from 100 msec pre-stimulus to 900 msec post-stimulus. The recorded EEG was digitally filtered offline using a band-pass filter of 0.01-30 Hz for N100-P3b and of 1-30 Hz for MMN-P3a.

*Resting-state EEG indices*

For frequency bands analysis data were parsed into epochs of 2-s duration, recomputed to average reference and frequency transformed by means of a fast Fourier transform (FFT) with a 10% Hanning window. The 2-s epochs averages were based on a minimum of 50% total epochs for each EEG record. Spectral amplitude was computed as the square root of spectral power and averaged across the artifact free epochs of each subject using Brain Vision Analyzer software. Based on previous literature data, spectral amplitude was averaged within the following ten frequency bands: delta, 0.5–3.5 Hz; theta1, 3.5–6.5 Hz; theta2, 6.5–8.5 Hz; alpha1, 8.5–10.5 Hz; alpha2, 10.5–12.5 Hz; beta1, 12.5–18.5 Hz; beta2, 18.5–21.5 Hz; beta3, 21.5–30 Hz; low gamma, 30–40 Hz and upper gamma 40–70 Hz (1).

For microstates analysis, we use a MATLAB-based open source toolbox Randomization Graphical User interface (Ragu) (2). First, we extracted the individual MS parameters using a MS map template from a normative study (eTable2) (3). Individual EEG data were spatially down-sampled to 19 channels and all maps at momentary GFP peaks were assigned to the microstate template maps using a best-fit criterion (a Cross-Validation criterion which is a modified version of the predictive residual variance) (4, 5). We obtained the assignment of the time-points between GFP peaks, using a nearest neighbor interpolation. For each MS class, the mean duration and mean count (occurrence) per second were computed, as well as the relative time contribution (percentage of time covered by each MS class).

*ERPs*

ERPs were extracted in each subject by the averaging method, in order to improve the signal/noise ratio, ruling out baseline activity not related to the stimulus. N100 and P3b components for standard (ST) and target (TA) tones were analyzed separately. MMN and P3a waves were extracted on all the “deviant” trials separately for duration and pitch (respectively Duration Deviant - DD and Pitch Deviant - PD). Trials with drifts larger than ±100 μV for N100-P3b and ±75 μV for MMN-P3a in any scalp electrode were rejected. If following artifacts and noisy trials removal, less than 40 usable target trials for N100-P3b (50% of target trials) and of 100 usable trials for MMN-P3a (50% of d- or p-deviant trials) remained, the subject was excluded from the analysis. Data were baseline-corrected using the 100 msec time window preceding stimuli. N100 peak was the most negative point between 80-120 msec, and P3b peak was the most positive point from 240 to 480 msec after stimulus, in the signal obtained from the auditory “oddball” task. P3a was the most positive point between 230-380 msec after stimulus, in the signal obtained from the auditory pre-attentive task. For MMN analysis, peaks for duration deviant (dMMN), and pitch deviant (pMMN) tones were automatically marked as the most negative point ranging from 90-250 msec on the difference curves deriving from subtracting the standard tone waveform from the duration and pitch deviant ones. Due to their prevalent localization, peak amplitude and latency of the above-mentioned ERPs were analyzed from Cz, Fz and Pz electrodes.

- 1. Machine learning pipeline

We built a double cycle, nested cross-validation (CV) framework, to allow for unbiased estimation of models’ generalizability and to prevent information leakage between subjects used for training and testing the models. This nested CV structure implies the presence of two loops: an inner CV loop, where models are generated, embedded in a super-ordinate loop (outer CV loop) which is ultimately used to test the models’ generalizability. In the inner CV loop we first computed nearest neighbor-based imputation to fill missing values in the matrices, where missing data occurred, followed by training data scaling from 0 to 1 and pruning of zero-variance features. We employed a linear Support Vector Machine (SVM) to detect a set of features that optimally predicted the training and test cases’ labels in a given CV inner partition. We implemented hyperparameter optimization, involving testing ranges (and not single values) of the C (misclassification cost) parameter, within a grid defined by the ranges C = [0.0156 — 16]. In order to balance groups (the SCZ group was bigger than the control group) we undersampled the majority group so that the groups are balanced when the models are created in the CV inner folds.

For the optimization of the classifiers, we performed a greedy sequential forward wrapper approach at each SVM C-regularization parameter with early stopping at 20% of the features selected. The goal of this approach was to find a restricted feature set among the input variables that would most economically predict outcome labels, and hence would be more suitable for real-world application than the full feature set (6). This analysis pipeline was then applied to the outer CV loop determining the participants’ classification (SCZs vs. HCs). Both in inner (CV1) and outer (CV2) CV levels we employed a 10-fold CV cycle. We extended nested CV to repeated nested CV at both inner and outer cross-validation cycles by randomly permuting the participants within their groups (number of permutations = 10) and repeating the CV cycle for each of these permutations. To build the global classifier, we combined unimodal classifiers’ outputs within the machine learning environment through a “stacking” procedure (7). Stacking procedure is utilized to ensemble different machine learning algorithms. There are two kinds of models in a stacked generalization framework: several base models (level-0 models) and one meta-model (level-1 model). The essence of stacking procedure is to use the level-1 model to learn from the predictions of level-0 models. Generally, a stacked generalization framework can obtain more accurate prediction compared to the best level-0 model. The contribution of each level-0 model to the level-1 model was defined calculating the Spearman’s correlation between the decision scores of each level-0 model and the scores of the level-1 classifier.

1. **eResults**

2.1 Pearson’s correlations among the 10% most frequently selected features of each classifier

The analysis showed a strong direct relationship (p<0.001) among theta 1 and theta 2 activities measured at different sensors, among alpha 1 and 2 activities measured at different sensors, among DD MMN amplitudes, and among P300 amplitudes after target stimuli. The GFP-Peaks-AD and the occurrence AD showed a strong inverse correlation (p<0.001) with frequency band activity, in particular with theta bands activity. The duration AD showed a direct correlation (p<0.001) with theta 2 and alpha 1 activity and an inverse correlation (p<0.001) with GFP-Peaks-AD and occurrence AD.

1. **eTables**

Table 1 Supplement. Changes over time of the four Non-Negative Matrix Factorization (NNMF) factor scores

| **Factor** | **Baseline** | **Follow-up** | **t** | **p** |
| --- | --- | --- | --- | --- |
| Factor 1 – Positive symptoms and parkinsonism | 0.041 ± 0.042 | 0.034 ± 0.040 | 1.20 | 0.24 |
| Factor 2 – Negative symptoms | 0.046 ± 0.034 | 0.049 ± 0.040 | -0.44 | 0.66 |
| Factor 3 – Depression | 0.055 ± 0.061 | 0.043 ± 0.062 | 1.18 | 0.24 |
| Factor 4 – Functioning and cognitive disturbances | 0.060 ± 0.033 | 0.072 ± 0.041 | -2.91 | 0.01 |

Table 2 Supplement. Classification performance (SCZs vs HCs) of Machine Learning models (all the EEG features together) without and with gender among predictors.

| **Classification**  **SCZs vs HC** | **Number of variables** | **TN** | **TP** | **FN** | **FP** | **Sensitivity** | **Specificity** | **Balanced**  **Accuracy** | **PPV** | **NPV** | **NND** | **PLR** | **Diagnostic odds ratio** | **P value** |
| --- | --- | --- | --- | --- | --- | --- | --- | --- | --- | --- | --- | --- | --- | --- |
| **EEG** | 397 | 46 | 90 | 23 | 11 | 79.6 | 80.7 | 80.2 | 89.1 | 66.7 | 1.7 | 4.1 | 17.0 | <0.001 |
| **EEG with Gender** | 398 | 46 | 92 | 21 | 11 | 81.4 | 80.7 | 81.1 | 89.3 | 68.7 | 1.6 | 4.2 | 17.8 | <0.001 |

Table 3 Supplement. Microstates (MS) parameters

| GFP Peaks A | Global Field Power – Peak – MS-A |
| --- | --- |
| GFP Peaks B | Global Field Power – Peak – MS-B |
| GFP Peaks C | Global Field Power – Peak – MS-C |
| GFP Peaks D | Global Field Power – Peak – MS-D |
| GFP Peaks AD | Sum of Global Field Power – Peaks of MS-A, MS-B, MS-C, MS-D |
| Contrib A | Percentage of time covered by MS-A |
| Contrib B | Percentage of time covered by MS-B |
| Contrib C | Percentage of time covered by MS-C |
| Contrib D | Percentage of time covered by MS-D |
| Occur A | Mean number of occurrence of MS-A |
| Occur B | Mean number of occurrence of MS-B |
| Occur C | Mean number of occurrence of MS-C |
| Occur D | Mean number of occurrence of MS-D |
| Occur AD | Sum of the mean number of occurrence of MS-A, MS-B, MS-C, MS-D |
| Dur A | Duration of MS-A |
| Dur B | Duration of MS-B |
| Dur C | Duration of MS-C |
| Dur D | Duration of MS-D |
| Dur AD | Mean of the duration of MS-A, MS-B, MS-C, MS-D |
| TM 0gt1 | Transition from MS-A to MS-B |
| TM 0gt2 | Transition from MS-A to MS-C |
| TM 0gt3 | Transition from MS-A to MS-D |
| TM 1gt0 | Transition from MS-B to MS-A |
| TM 1gt2 | Transition from MS-B to MS-C |
| TM 1gt3 | Transition from MS-B to MS-D |
| TM 2gt0 | Transition from MS-C to MS-A |
| TM 2gt1 | Transition from MS-C to MS-B |
| TM 2gt3 | Transition from MS-C to MS-D |
| TM 3gt0 | Transition from MS-D to MS-A |
| TM 3gt1 | Transition from MS-D to MS-B |
| TM 3gt2 | Transition from MS-D to MS-C |
| Delta AgtB | Delta between MS-A and MS-B |
| Delta AgtC | Delta between MS-A and MS-C |
| Delta AgtD | Delta between MS-A and MS-D |
| Delta BgtA | Delta between MS-B and MS-A |
| Delta BgtC | Delta between MS-B and MS-C |
| Delta BgtD | Delta between MS-B and MS-D |
| Delta CgtA | Delta between MS-C and MS-A |
| Delta CgtB | Delta between MS-C and MS-B |
| Delta CgtD | Delta between MS-C and MS-D |
| Delta DgtA | Delta between MS-D and MS-A |
| Delta DgtB | Delta between MS-D and MS-B |
| Delta DgtC | Delta between MS-D and MS-C |

Table 4 Supplement. Group comparisons (SCZs vs HCs) on the 10% most selected features of each classifier

| **EEG Index** | **SCZ (mean±SD)** | **HC (mean±SD)** | **t(df)** | **p** | **Cohen’s d** |
| --- | --- | --- | --- | --- | --- |
| Fp1 Theta 1 | 0.424±0.168 | 0.350±0.118 | 2.984 (168) | .001 | 0.485 |
| Fp2 Theta 1 | 0.424±0.157 | 0.345±0.116 | 3.332 (168) | <.001 | 0.541 |
| T3 Theta 1 | 0.397±0.162 | 0.352±0.137 | 1.806 (168) | .073 | 0.293 |
| Pz Theta 1 | 0.443±0.163 | 0.358±0.126 | 3.474 (168) | .001 | 0.564 |
| O2 Theta 1 | 0.499±0.244 | 0.383±0.154 | 3.266 (168) | <.001 | 0.531 |
| T6 Theta 1 | 0.491±0.225 | 0.381±0.158 | 3.294 (168) | <.001 | 0.535 |
| MSD Theta 1 | 0.485±0.189 | 0.404±0.139 | 2.869 (168) | .002 | 0.466 |
| Fp1 Theta 2 | 0.494±0.250 | 0.358±0.187 | 3.602 (167) | <.001 | 0.589 |
| Fp2 Theta 2 | 0.492±0.251 | 0.359±0.196 | 3.495 (168) | <.001 | 0.568 |
| Fpz Theta 2 | 0.491±0.255 | 0.358±0.200 | 3.418 (168) | <.001 | 0.555 |
| AF3 Theta 2 | 0.500±0.254 | 0.378±0.178 | 3.255 (168) | <.001 | 0.529 |
| F3 Theta 2 | 0.488±0.239 | 0.373±0.157 | 3.287 (168) | <.001 | 0.534 |
| P4 Theta 2 | 0.540±0.301 | 0.376±0.199 | 3.721 (168) | <.001 | 0.605 |
| PO7 Theta 2 | 0.660±0.397 | 0.461±0.262 | 3.441 (168) | <.001 | 0.559 |
| PO8 Theta 2 | 0.699±0.413 | 0.478±0.284 | 3.630 (168) | <.001 | 0.590 |
| O1 Theta 2 | 0.607±0.341 | 0.423±0.234 | 3.664 (168) | <.001 | 0.595 |
| O2 Theta 2 | 0.602±0.327 | 0.417±0.240 | 3.792 (168) | <.001 | 0.616 |
| Oz Theta 2 | 0.567±0.328 | 0.394±0.227 | 3.589 (168) | <.001 | 0.583 |
| T5 Theta 2 | 0.621±0.354 | 0.448±0.252 | 3.292 (168) | <.001 | 0.535 |
| T6 Theta 2 | 0.669±0.382 | 0.452±0.265 | 3.849 (168) | <.001 | 0.625 |
| MSS Theta 2 | 0.554±0.233 | 0.411±0.196 | 3.965 (168) | <.001 | 0.644 |
| MSD Theta 2 | 0.561±0.257 | 0.414±0.187 | 3.829 (168) | <.001 | 0.622 |
| PO7 Alpha 1 | 0.938±0.569 | 0.957±0.743 | -0.182 (168) | .868 | -0.030 |
| C4 Alpha 2 | 0.427±0.240 | 0.568±0.387 | -2.939 (168) | .013 | -0.478 |
| Cz Alpha 2 | 0.438±0.266 | 0.565±0.361 | -2.583 (168) | .021 | -0.420 |
| P3 Alpha 2 | 0.580±0.319 | 0.751±0.538 | -2.603 (168) | .030 | -0.423 |
| MSD Alpha 2 | 0.450±0.195 | 0.549±0.327 | -2.457 (168) | .039 | -0.399 |
| Oz Beta 1 | 0.340±0.181 | 0.292±0.142 | 1.721 (168) | .087 | 0.280 |
| Fpz Beta 3 | 0.178±0.072 | 0.166±0.046 | 1.125 (168) | .198 | 0.183 |
| GFP-Peaks-AD | 24.265±3.444 | 25.336±2.839 | -2.025 (168) | .044 | -0.329 |
| Occurrence AD | 16.407±3.195 | 16.803±3.442 | -0.742 (168) | .459 | -0.121 |
| Duration AD | 60.770±10.870 | 59.750±11.610 | 0.564 (168) | .573 | 0.092 |
| Delta BgtC | 0.002±0.009 | 0.0001±0.007 | 1.853 (168) | .066 | 0.301 |
| Delta BgtD | -0.004±0.006 | -0.002±0.008 | -1.664 (168) | .098 | -0.270 |
| Fz-MMN-DD-amplitude | -3.464±1.894 | -5.565±2.460 | 6.158 (168) | <.001 | 1.000 |
| Cz-MMN-DD-amplitude | -3.131±1.666 | -4.832±2.253 | 5.562 (168) | <.001 | 0.904 |
| Pz-MMN-DD-amplitude | -1.782±1.198 | -2.683±1.357 | 4.426 (168) | <.001 | 0.719 |
| Pz-P3a-DF-amplitude | 0.603±0.836 | 1.069±0.904 | -3.339 (168) | .001 | -0.542 |
| TA-P300-Fz-amplitude | 4.258±5.276 | 7.806±5.179 | -4.166 (168) | <.001 | -0.677 |
| TA-P300-Pz-amplitude | 9.332±7.235 | 14.018±6.048 | -4.204 (168) | <.001 | -0.683 |

1. **Figures**

Figure 1 Supplement. Correlation matrix plot among the 10% most selected features of each classifier. The colormap on the right indicates the values of Pearson’s correlation index (r is between -1 and 1).


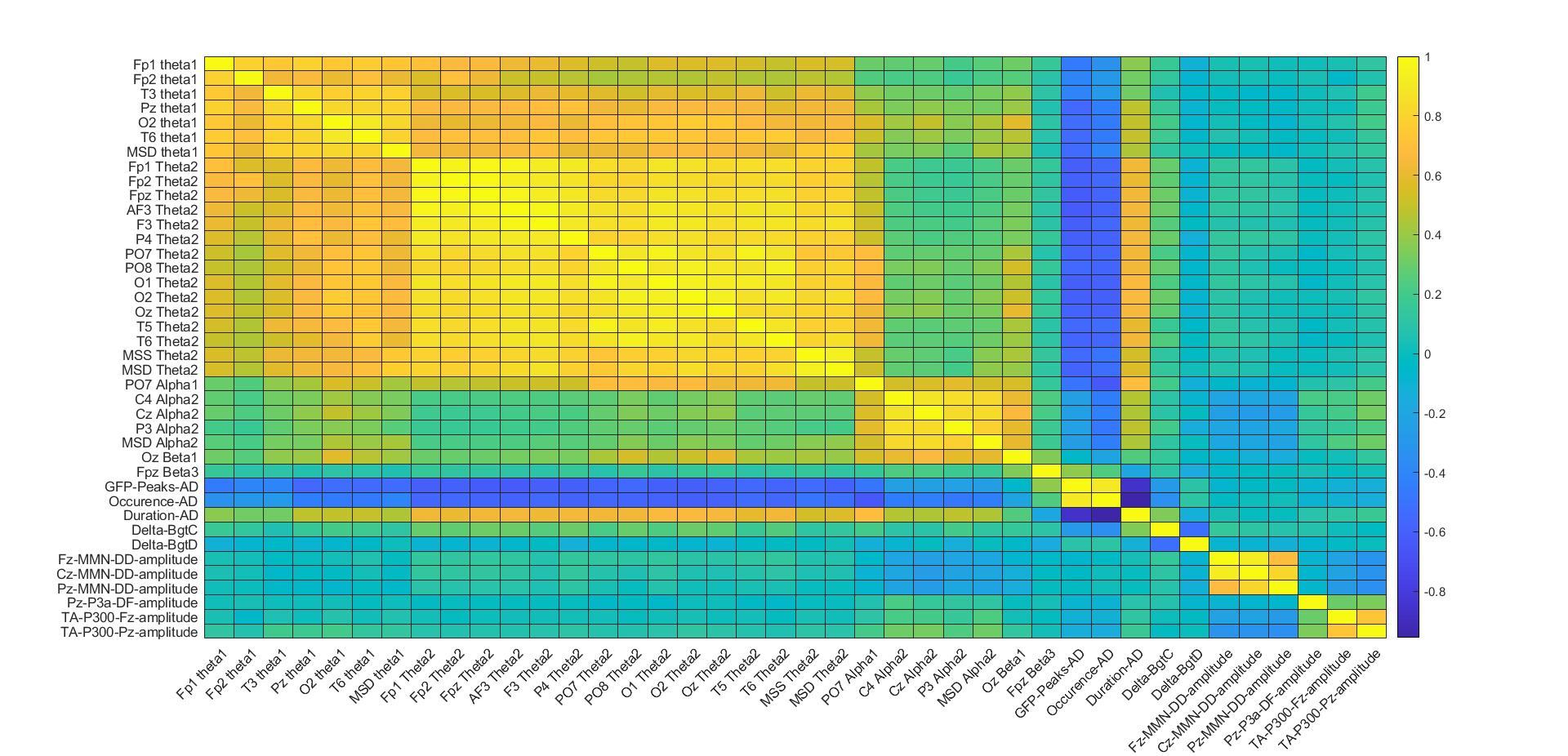


1. **eReferences**
2. Koenig T, Lehmann D, Merlo MC, Kochi K, Hell D, Koukkou M. A deviant EEG brain microstate in acute, neuroleptic-naive schizophrenics at rest. Eur Arch Psychiatry Clin Neurosci. 1999;249(4):205-11.
3. Habermann M, Weusmann D, Stein M, Koenig T. A Student's Guide to Randomization Statistics for Multichannel Event-Related Potentials Using Ragu. Front Neurosci. 2018 Jun 19;12:355. doi: 10.3389/fnins.2018.00355.
4. Koenig T, Prichep L, Lehmann D, Sosa PV, Braeker E, Kleinlogel H, et al. Millisecond by millisecond, year by year: normative EEG microstates and developmental stages. Neuroimage. 2002;16(1):41-8.
5. Murray MM, Brunet D, Michel CM. Topographic ERP analyses: a step-by-step tutorial review. Brain Topogr. 2008 Jun;20(4):249-64. doi: 10.1007/s10548-008-0054-5.
6. Pascual-Marqui RD, Michel CM, Lehmann D. Segmentation of brain electrical activity into microstates: model estimation and validation. IEEE Trans Biomed Eng. 1995 Jul;42(7):658-65. doi: 10.1109/10.391164.
7. Koutsouleris N, Dwyer DB, Degenhardt F, Maj C, Urquijo-Castro MF, Sanfelici R, et al. Multimodal Machine Learning Workflows for Prediction of Psychosis in Patients With Clinical High-Risk Syndromes and Recent-Onset Depression. JAMA Psychiatry. 2021;78(2):195-209.
8. Ma Z, Wang P, Gao Z, Wang R, Khalighi K. Ensemble of machine learning algorithms using the stacked generalization approach to estimate the warfarin dose. PLoS One. 2018;13(10):e0205872.
